# The Prognostic Role of Advanced Lung Cancer Inflammation Index in Patients with Idiopathic Pulmonary Fibrosis

**DOI:** 10.3390/jcm13195874

**Published:** 2024-10-02

**Authors:** Fulsen Bozkuş, Olgun Keskin

**Affiliations:** Department of Chest Disease, University of Health Sciences, Antalya Training and Research Hospital, 07050 Antalya, Turkey

**Keywords:** idiopathic pulmonary fibrosis, advanced lung cancer inflammation index, albumin, body mass index, neutrophil-to-lymphocyte ratio

## Abstract

**Background**: The advanced lung cancer inflammation index (ALI) is an innovative and thorough measure designed to assess both inflammation and nutritional status. It includes parameters such as albumin, body mass index (BMI), and the neutrophil-to-lymphocyte ratio (NLR). This research seeks to evaluate the prognosis of idiopathic pulmonary fibrosis (IPF) patients by integrating both inflammation and nutritional status, distinguishing it from conventional inflammation biomarkers. **Methods**: This study included 102 patients with IPF. Clinical data were extracted from the patients’ medical records. NLR and ALI scores were calculated based on data collected at the initiation of antifibrotic treatment using the following formulas: Neut/Lym for NLR and albumin × BMI/NLR for ALI. **Results**: ALI values were assessed across various IPF patient subgroups based on gender–age–physiology (GAP) stages (1, 2, and 3), forced vital capacity (FVC) (median split: <70% vs. ≥70%), diffusing capacity for carbon monoxide (DLCO) (<51% vs. ≥51%), 6-Minute Walk Test (6MWT) (<350 vs. ≥350), and the Charlson comorbidity index (CCI) (≤1 vs. >1). Significant differences in ALI were observed with respect to GAP stages, FVC, DLCO, and 6MWT categories (*p* = 0.000 for all), but not for CCI categories (*p* = 0.233). Receiver operating characteristic (ROC) curve analysis revealed that ALI had a sensitivity of 63.6% and a specificity of 98.9% at a threshold of 11.2 (AUC = 0.945, 95% CI 0.892–0.998, *p* < 0.000). **Conclusions**: Our findings indicate that ALI levels are significantly associated with disease severity and mortality in IPF patients.

## 1. Background

Idiopathic pulmonary fibrosis (IPF) is a chronic, progressive lung disease of unknown origin, characterized by severe fibrosis and a generally poor prognosis. The mortality rate for IPF ranges from approximately 0.5 to 12 deaths per 100,000 individuals, with patients typically experiencing a limited survival time [1].

The progression of IPF can be unpredictable, with some cases exhibiting a rapid decline while others progress more slowly. Additionally, patients may experience exacerbations that can lead to a shorter survival time. Various lung function parameters have been proposed to differentiate between patients with differing prognoses, and there are also scores that integrate clinical, functional, radiological, and hematological data [2,3,4]. Recently, there has been growing interest in inflammation indices derived from blood cell measurements. Although recent studies suggest that inflammatory cells play a role in various stages of IPF pathogenesis, there is ongoing debate about whether inflammation is a causative factor or merely a consequence of the fibrotic process [5].

The advanced lung cancer inflammation index (ALI) is an innovative and comprehensive metric that integrates factors such as albumin, body mass index (BMI), and the neutrophil-to-lymphocyte ratio (NLR) to assess both inflammation and nutritional status. Originally developed to predict prognosis in cancer patients, this index has recently been explored for its relevance in various inflammatory conditions, including heart failure, coronary artery disease, hypertension, and diabetes, due to its ability to concurrently evaluate inflammation and nutritional status [6]. Studies have shown that a decreased ALI value (i.e., low BMI and albumin, high NLR) reflects poorer nutritional status and a more severe inflammatory response in patients [7,8]. ALI aids in evaluating the overall inflammatory state and nutritional status of patients, providing insights into disease severity and potentially guiding treatment decisions. Consequently, assessing the prognosis of IPF patients based solely on inflammatory markers may be insufficient, and a comprehensive evaluation incorporating clinical indicators such as albumin and BMI is necessary. While BMI and albumin, key indicators of nutritional status, are recognized clinical markers for assessing malnutrition in IPF patients, their significance has not been fully appreciated [9]. Previous research has indicated that low BMI and weight loss may predict a rapid decline in lung function [10]. To our knowledge, no research has yet examined the prognostic significance of ALI in IPF patients. This study aims to evaluate the prognostic value of ALI and propose its potential utility in predicting mortality.

## 2. Materials and Methods

A retrospective review was conducted on the medical records of 123 patients diagnosed with IPF at Antalya Training and Research Hospital from June 2020 to July 2024. The diagnosis of IPF was confirmed using high-resolution computed tomography, which identified either the usual interstitial pneumonia (UIP) pattern or a possible UIP pattern, in accordance with the 2022 international consensus guidelines, which exclude other interstitial lung diseases or overlapping conditions [11]. All study participants were over 18 years old, and their clinical findings, complete blood count results, and albumin levels were recorded within 24 h of hospital admission. Biochemical tests included albumin, ALT (alanine aminotransferase), AST (aspartate aminotransferase), and LDH (lactate dehydrogenase enzyme), which were analyzed using the Beckman Coulter AU5800 (Beckman Coulter Inc., Brea, CA, USA) autoanalyzer with Beckman Coulter commercial kits through a spectrophotometric method. The hemogram (complete blood analysis) was performed on the Sysmex XT-2000i (Sysmex, Kobe, Japan). Patients with incomplete data, serious life-threatening diseases unrelated to IPF, malignancies, bleeding tendencies, severe liver or kidney dysfunction, or those who had used immunosuppressive drugs or oral corticosteroids in the past 3 months were excluded from this study. Consequently, 21 patients were excluded, resulting in a final analysis cohort of 102 patients.

Clinical data were obtained from the patients’ medical records. Laboratory results and pulmonary function test (PFT) outcomes were documented from the initiation of antifibrotic treatment. The gender–age–physiology (GAP) score for diagnosis and the Charlson comorbidity index (CCI), which assesses comorbidities that could impact mortality risk, were calculated as described in previous studies [12,13]. The date of the PFT was used as the date of diagnosis for each patient. All diagnosed patients were enrolled in the adult vaccination program. The observation period extended from the start of antifibrotic treatment to the final visit, with follow-up concluding in July 2024. Survival duration was defined as the time from the date of the spirometry test to either the date of death or, for patients still alive, until July 2024. In cases where the final status was not available in the records, phone calls were made to confirm survival status. It was observed that, during the observation period, although patients were referred to a pulmonary rehabilitation center, they were unable to undergo rehabilitation due to the absence of such a facility in their locality, which impacted their access to the service. For patients diagnosed with IPF, for whom antifibrotic treatment decisions were made, the NLR, BMI, and albumin values at the start of treatment were used to calculate the ALI. NLR and ALI were assessed at a single time point, with the scores calculated using the following formulas: Neutrophils/Lymphocytes and (albumin × BMI)/NLR. At the time of peripheral leukocyte assessment, none of the patients had active infections.

This study was approved by the Antalya Training and Research Hospital Human Research Ethics Committee on 13 June 2024, under decision number 9/16, and was conducted in accordance with the Helsinki Declaration. Given its retrospective design, this study was exempt from the requirement for informed consent.

## 3. Statistical Analysis

Statistical analysis was conducted using SPSS version 23.0. The normality of variable distributions was assessed through visual methods (such as histograms and probability plots) and statistical tests (Kolmogorov–Smirnov and Shapiro–Wilk tests). For variables with normal distributions, descriptive statistics are reported as means and standard deviations, whereas medians and interquartile ranges (IQRs) are used for variables with non-normal distributions. Differences between groups were examined using *t*-tests or Mann–Whitney U tests for continuous variables, and ANOVA or Kruskal–Wallis H tests as appropriate. Categorical variables were summarized using frequencies and percentages, with group differences assessed using the chi-square test. Correlations between variables were analyzed using Pearson or Spearman correlation coefficients. The predictive value of the advanced lung cancer inflammation index (ALI) and neutrophil-to-lymphocyte ratio (NLR) levels for mortality was evaluated using receiver operating characteristic (ROC) curve analysis, with sensitivity and specificity determined for significant cutoff points. Statistical significance was defined as *p*-values less than 0.05, with a type I error rate of less than 5% considered statistically significant.

## 4. Results

### 4.1. Baseline Characteristics of the Study Population

Table 1 provides an overview of the demographic and clinical characteristics of the 102 patients with IPF. The average age of the cohort at diagnosis was 70.4 ± 7.39 years, with an average BMI of 24.1 ± 1.79 kg/m^2^. The cohort consisted of 78.4% male participants, with the majority being former smokers (71.6%). According to GAP stages, 44 patients (43.1%) were classified in GAP stage 1, 32 patients (31.4%) in GAP stage 2, and 26 patients (25.5%) in GAP stage 3. In the study cohort, 53 cases (52%) were receiving nintedanib, while 49 cases (48%) were receiving pirfenidone. The mean observation period was 33.4 ± 8.61 months, during which 11 patients (10.8%) died (Table 1).

We also compared IPF subgroups in terms of ALI across GAP stages (1, 2, and 3), forced vital capacity (FVC) (median split, <70% vs. ≥70%), diffusing capacity for carbon monoxide (DLCO) (<51% vs. ≥51%), 6-Minute Walk Test (6MWT) (<350 vs. ≥350), and CCI (≤1 vs. >1). ALI showed significant differences based on GAP stages, FVC, DLCO, and 6MWT, but these differences were not observed for CCI categories or antifibrotic use (respectively, *p* = 0.000, *p* = 0.000, *p* = 0.000, *p* = 0.001, *p* = 0.233 for CCI, *p* = 0.150) for antifibrotic use (Table 2).

Additionally, significant differences in NLR, BMI, and albumin levels were observed when individuals were grouped according to GAP stages (*p* = 0.000 for all). Significant differences were also observed when ALI levels were categorized into Quantile 1 (<21.2), Quantile 2 (21.3–31.4), and Quantile 3 (>31.5). Notable differences in albumin, BMI, and NLR values were observed between the Quantile 1 and Quantile 2 groups (*p* = 0.000), Quantile 1 and Quantile 3 groups (*p* = 0.000), and Quantile 2 and Quantile 3 groups (*p* = 0.000 for albumin, *p* = 0.000 for BMI, and *p* = 0.000 for NLR). However, these significant differences were not observed based on antifibrotic use (Table 2).

### 4.2. Association between ALI, GAP, NLR, BMI, FVC, DLCO, 6MWT, and Albumin

The Pearson correlation analysis showed a strong negative correlation between ALI and the GAP stage (r = −0.815, *p* < 0.0001). In contrast, ALI exhibited significant positive correlations with FVC (r = 0.498, *p* < 0.0001), DLCO (r = 0.637, *p* < 0.0001), and the 6MWT (r = 0.445, *p* < 0.0001). Albumin and BMI also exhibited similar correlations with the GAP stage and lung function parameters to ALI. Specifically, as the GAP stage increased, ALI, BMI, and albumin values decreased, while FVC, DLCO, and 6MWT also decreased. The analysis revealed that NLR had a significant positive correlation with the GAP stage (r = 0.638, *p* < 0.0001) and significant negative correlations with both FVC (r = −0.348, *p* < 0.0001) and DLCO (r = −0.525, *p* < 0.0001) (see Table 3).

### 4.3. Cutoff Values of ALI Components for Predicting Mortality Risk

**Figure 1:**
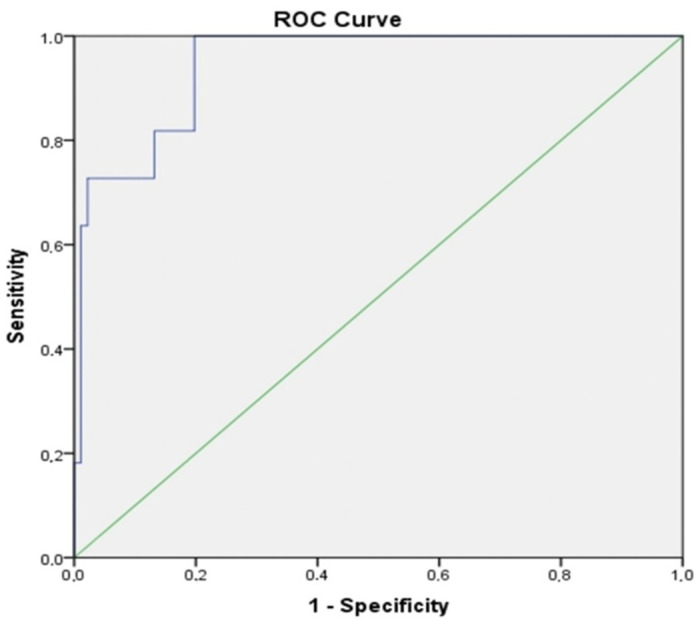
ROC curve of ALI for predicting mortality risk. Green line: reference line; Blue line: ALI.

ROC curve analysis was conducted to assess the sensitivity and specificity of the parameters constituting ALI in predicting mortality risk in IPF patients. All area under the curve (AUC) values were found to be significant (Table 4). The best-performing index was albumin, with a threshold of 2.45, demonstrating 63.6% sensitivity and 97.8% specificity (AUC = 0.952, 95% CI 0.904–1.000, *p* < 0.001). Similar to albumin, ALI showed 63.6% sensitivity and 98.9% specificity at a threshold of 11.2, with an AUC of 0.945 (95% CI 0.892–0.998, *p* < 0.0001) (Figure 1). When comparing the median ALI values between survivors and non-survivors, it was observed that survivors had a median ALI of 28.4 (IQR: 16.84), whereas non-survivors had a median ALI of 11.0 (IQR: 8.83), revealing a statistically significant difference (*p* < 0.0001). Consequently, ROC analysis was used to assess the predictive capability of ALI for mortality risk. However, no statistically significant correlation was found between ALI values and patients’ survival durations (r = 0.181, *p* = 0.068).

## 5. Discussion

To our knowledge, this is the first retrospective study to evaluate the ALI in patients with IPF undergoing antifibrotic treatment, incorporating both inflammation and nutritional status. Our findings reveal statistically significant differences in ALI levels when patients were categorized by GAP stages and functional parameters. Importantly, ALI, along with albumin, NLR, and BMI, has been identified as a predictor of mortality in this cohort.

The fibrotic process in IPF is commonly understood as an aberrant healing response following injury to alveolar epithelial cells, leading to chronic inflammation and eventual fibrosis. While inflammation is thought to contribute to the development and progression of pulmonary fibrosis, recent evidence suggests that fibroblast dysfunction may play a more central role in the pathophysiology of IPF than persistent inflammation. Thus, inflammation may be more of an epiphenomenon rather than a primary driver of IPF [14].

ALI includes NLR as an inflammation marker. Neutrophils, activated by the immune response, perform various specialized functions and, when excessively activated, can exacerbate chronic inflammation and support both innate and adaptive immune responses [15]. Research indicates that all stages of fibrosis in IPF involve both innate and adaptive immune responses [16]. Elevated neutrophil counts are indicative of non-specific inflammation, while reduced lymphocyte counts suggest impaired immunity. Therefore, NLR reflects an individual’s immunological status and inflammatory response [17]. In our study, the mean NLR in patients with IPF was 3.3, which is higher than the average levels reported in healthy individuals (1.65–2.11) [18,19]. However, NLR values can vary depending on the methods and populations studied, and a universal reference value has yet to be established.

Previous studies have demonstrated that the NLR is a reliable marker of systemic inflammation in IPF and is significantly associated with functional lung parameters [20]. Additionally, research assessing NLR in bronchoalveolar lavage (BAL) fluid has revealed a negative correlation between NLR and both FVC and FEV1, as well as an association with the composite physiological index measured during BAL sample collection [21]. These findings are consistent with research suggesting that NLR can serve as a prognostic indicator in various chronic inflammatory diseases [22,23,24,25]. Our study also identified a significant negative correlation between NLR and both FVC and DLCO, reinforcing previous results that a high NLR is associated with poor prognosis in IPF patients.

The GAP model, which incorporates age, sex, FVC, and DLCO, is a well-established tool for predicting prognosis in IPF and other chronic interstitial lung diseases [12]. Recent research has explored enhancing the GAP model by integrating peripheral leukocyte analyses to improve mortality risk prediction. For instance, one study found that combining the GAP model with the NLR effectively stratified mortality risk, with a median NLR ≥ 2.9 being associated with increased mortality [26]. Another study developed a risk scoring system that integrated the CCI and monocyte levels into the GAP model for IPF patients. This research indicated that patients with lower FVC, reduced DLCO, more comorbidities, and higher monocyte levels had the poorest survival outcomes [27]. Furthermore, combining the GAP index and NLR effectively classified mortality risk both at the initiation of antifibrotic treatment and during long-term follow-up. However, subgroup analyses indicated that NLR was less effective in advanced-stage disease or non-IPF patients [28]. In our study, although we did not specifically evaluate the GAP model and CCI in conjunction with the ALI, we observed statistically significant differences in NLR and ALI values when cases were categorized according to GAP stages. Our findings revealed a notable negative correlation between the GAP stage and ALI, indicating that as the GAP stage increases, ALI tends to decrease. Conversely, there was a significant positive correlation between the GAP stage and NLR, suggesting that higher GAP stages are associated with higher NLR values. This implies that as the GAP stage advances, NLR levels increase while ALI values decrease. No significant differences were observed when cases were categorized based on the CCI.

Studies examining the relationship between the NLR and mortality in IPF and idiopathic pleuropulmonary fibroelastosis suggest that NLR may reflect disease severity regardless of the timing of assessment. Research has shown that NLR levels remain relatively stable one year after treatment initiation, with persistently high NLR levels correlating with an increased risk of mortality. These findings underscore the role of NLR in the progression of pulmonary fibrosis [29,30]. In our study cohort, NLR and ALI were assessed at a single time point, with high NLR and low ALI being associated with an increased mortality risk. ROC analysis revealed an AUROC for NLR in predicting mortality risk was 0.768, while the AUROC for ALI was 0.945. These results suggest that both NLR and ALI have the potential to serve as prognostic biomarkers for predicting mortality risk in patients with IPF.

Albumin, a key component of ALI, is influenced by inflammation and malnutrition, often leading to reduced albumin levels and weight loss. As a sensitive marker of nutritional status, lower albumin levels have been associated with shorter survival times in patients with IPF [31]. In our study, albumin levels were significantly lower in the Quantile 1 (low ALI) group when ALI values were analyzed by percentiles. We observed a significant positive correlation between ALI and albumin levels. ROC analysis revealed that the AUROC for albumin in predicting mortality was 0.952, indicating a strong relationship between albumin levels and mortality risk in IPF patients. Consistent with these findings, lower albumin levels were associated with increased mortality risk. Our study demonstrates that the AUROC value for albumin in predicting mortality risk is higher than that for ALI, which corroborates the existing literature. These results suggest that, consistent with the literature, ALI values, like albumin, can also be utilized as a parameter for predicting mortality risk.

In patients with advanced lung disease, malnutrition is strongly associated with the development of sarcopenia and cachexia, conditions that are prevalent in these cases [32,33]. Recent studies have demonstrated that malnutrition increases the risk of hospitalization and mortality in patients with IPF. BMI, a component of ALI, serves as a crucial clinical indicator for evaluating nutritional status and prognosis in IPF patients, independent of antifibrotic therapy [34,35]. Analysis of the placebo group from the INPULSIS study, when stratified by BMI below and above 25 kg/m^2^, demonstrated that the reduction in FVC was statistically significant. Additionally, patients who lost more than 5% of their body weight had a greater decline in FVC compared to those with less than 5% weight loss. This suggests that low BMI and significant weight loss are important clinical indicators of poor prognosis in IPF [7]. Research assessing malnutrition risk in IPF patients, based on serum albumin levels, body weight, and ideal body weight, found that this risk is an independent prognostic factor. This finding was consistent regardless of patients’ age, sex, FVC, or GAP index. Those at risk of malnutrition had markedly shorter survival times than those without this risk, both in isolated and pooled groups [36]. In our study, individuals with low BMI had lower ALI values, and there was a significant positive correlation between BMI and ALI. ROC analysis results indicate that the AUROC for BMI in predicting mortality risk is 0.885. This finding suggests that, similar to ALI, a low BMI may indicate an increased mortality risk in patients with IPF. These results highlight the critical role of nutritional status in the progression and outcomes of IPF, regardless of sex, age, or lung physiology. Our results suggest that ALI assessments could be a valuable tool for evaluating disease severity, prognosis, and mortality risk in IPF patients.

We found a significant relationship between ALI levels and both disease severity and mortality in IPF patients. While disease severity is typically assessed using physiological parameters such as FVC and/or DLCO, these measures have limitations—they can be influenced by patient effort, may be challenging to perform in critically ill patients, and might not always accurately predict mortality. In contrast, routine peripheral blood parameters are more accessible and can help overcome these limitations. Our study underscores the prognostic value of ALI, a simple and routinely measured biomarker, suggesting that evaluating both inflammation and nutritional status is crucial for a comprehensive assessment of IPF prognosis.

This study has limitations. The small sample size and single-center design may affect the generalizability of the results, especially given the rarity of IPF. Additionally, the cross-sectional nature of this study limits our ability to establish a definitive causal relationship between the investigated indices and IPF or pulmonary function parameters. Future research with larger sample sizes and prospective designs is needed to better clarify causality.

Despite using various statistical techniques to minimize bias, completely eliminating unknown confounding factors is challenging. As a retrospective observational study, it may be subject to selection bias and missing data. This study focused on patients without serious, life-threatening conditions, liver or kidney dysfunction, infections, or confirmed lung cancer prior to admission, potentially excluding a broader range of IPF patients. Additionally, the data were limited to those presenting at the hospital, with many subjects having only basic information available.

Additionally, BMI reference values used to calculate ALI vary by race and country. While the average BMI in Western countries is approximately 26–28 kg/m^2^, in Asian countries, it is around 23–24 kg/m^2^. Thus, ALI may not be universally applicable across all populations. Although our data indicate that low ALI levels are associated with an increased risk of mortality in patients, the small sample size limited our ability to perform stratified analyses.

In recent years, there has been growing interest in identifying indices that can accurately and reproducibly predict clinically important outcomes in IPF. ALI, which has been used to assess prognosis in cancer patients, evaluates both inflammation and nutritional status and has also proven useful in chronic inflammatory diseases. This study provides evidence supporting the use of ALI parameters for assessing prognosis in IPF patients. In summary, assessing prognosis is crucial for patient counseling, disease management, and lung transplantation guidance. Our research is the first to show that a decrease in ALI is closely associated with increased mortality risk in IPF patients. These findings highlight the importance of managing ALI within an appropriate range to improve long-term survival in IPF patients, through weight control, maintaining normal albumin levels, and antifibrotic treatments. Monitoring dynamic changes in ALI could help clinics establish personalized standards, potentially enhancing the long-term survival of IPF patients. ALI represents a new, simple, and robust prognostic biomarker in IPF, offering ease of use, accessibility, and cost-effectiveness.

## Figures and Tables

**Table 1:** Characteristics of the study population.

Variables	
Gender, M/F (*n*, %)	80 (78.4)/22 (21.6)
Age, years	70.4 ± 7.39
Smokers, never/current/ex (*n*, %)	13 (12.7)/16 (15.7)/73 (71.6)
BMI (kg/m^2^)	24.1 ± 1.79
Disease duration (months)	33.4 ± 8.61
AE (*n*, %)	15 (14.7)
Charlson comorbidity index	1.4 ± 0.62
GAP Stages	
I	44 (43.1)
II	32 (31.4)
III	26 (25.5)
GAP index (1/2/3/4/5/6/7)	2 (2.0)/12 (11.8)/30 (29.4)/16 (15.7)/16 (15.7)/19 (18.6)/7 (6.9)
Pulmonary function test	
FVC, %-pred	70.2 ± 7.54
FEV1, %-pred	74.7 ± 7.43
DLCO, %	49.7 ± 11.82
6MWT (meters)	359.2 ± 49.42
Laboratory variables	
Neutrophils (10^9^/L)	5.50 ± 1.26
Lymphocytes (10^9^/L)	1.7 ± 0.35
Monocytes (10^9^/L)	1.7 ± 0.35
Albumin, g/dL	3.5 ± 0.69
LDH, U/L	212.9 ± 65.90
ALT (U/L)	22.8 ± 28.70
AST (U/L)	20.8 ± 7.11
NLR	3.3 ± 1.05
ALI	29.6 ± 15.32
Survivor/non-survivor (*n*/%)	91 (89.2)/11 (10.8)
Nintedanib/pirfenidone (*n*/%)	53 (52.0)/49 (48.0)

Data are expressed as mean ± standard deviation, median (interquartile range), or *n* (%). Abbreviations: BMI: body mass index; AE: acute exacerbation; GAP: gender, age, and two lung physiology variable index; FVC: forced vital capacity; DLCO: diffusion capacity for carbon monoxide; 6MWT: six-minute walk test; AST: aspartate aminotransferase; ALT: alanine aminotransferase; LDH: lactate dehydrogenase; NLR: neutrophil/lymphocyte ratio; ALI: advanced lung cancer inflammation index.

**Table 2:** Comparison of clinical parameters and blood cell count indices among the groups.

		ALI		
		*n*	Median (IQR)	*p*
GAP stages	1 (0–3)	44	38.5 (18.60) ^a^	**0.000**
	2 (4–5)	32	21.6 (7.35) ^b^	
	3 (6–8)	26	17.5 (10.72) ^c^	
FVC (median split)	<70	44	21.1 (9.58)	**0.000**
	≥70	58	31.3 (20.05	
DLCO	<51	49	20.3 (10.75)	**0.000**
	≥51	53	32.0 (20.04)	
6MWT (meters)	<350	36	19.6 (11.63)	**0.001**
	≥350	66	29.7 (17.65)	
Charlson comorbidity index	≤1	65	27.5 (19.96)	0.233
	>1	37	22.1 (12.59)	
Nintedanib (*n*/%)		53	24.7 (12.29)	0.150
Pirfenidone (*n*/%)		49	28.7 (23.31)	
	GAP stage 1 (*n* = 44)	GAP stage 1 (*n* = 44)	GAP stage 3 (*n* = 26)	
BMI	25.2 ± 1.30 ^a^	23.7 ± 1.50 ^b^	22.5 ± 1.42 ^c^	**0.000**
NLR	2.5 ± 0.71 ^a^	3.6 ± 0.70 ^b^	4.1 ± 1.15 ^b, c^	**0.000**
Albumin	4.0 ± 0.53 ^a^	3.36 ± 0.45 ^b^	2.93 ± 0.11 ^c^	**0.000**
Neutrophils (10^9^/L)	4.5 ± 0.84 ^a^	6.1 ± 0.76 ^b^	6.5 ± 1.08 ^b, c^	**0.000**
Lymphocytes (10^9^/L)	1,6 (0)	1.7 (0)	1.6 (0)	0.070
Monocytes (10^9^/L)	0.8 (0)	0.7 (0)	0.9 (0)	0.114
ALI	38 (16.59) ^a^	25.1 (7.43) ^b^	17.6 (4.27) ^c^	**0.000**
	ALI Quantile 1 [<21.2] (*n* = 36)	ALI Quantile 2 [3–31] (*n* = 33)	ALI Quantile 3 [>31.5] (*n* = 33)	
BMI	22.9 ± 1.38 ^a^	24.1 ± 1.73 ^b^	25.2 ± 1.43 ^c^	**0.000**
NLR	4.2 ± 0.96 ^a^	3.3 ± 0.45 ^b^	2.3 ± 0.56 ^c^	**0.000**
Albumin	2.8 ± 0.48 ^a^	3.7 ± 0.41 ^b^	4.0 ± 0.55 ^c^	**0.000**
Neutrophils (10^9^/L)	6.4 ± 0,96 ^a^	5.7 ± 0.86 ^b^	4.2 ± 0.76 ^c^	**0.000**
Lymphocytes (10^9^/L)	1.6 (0) ^a^	1.7 (0) ^b^	1.9 (1) ^b, c^	**0.000**
Monocytes (10^9^/L)	0.9 (0)	0.8 (0)	0.8 (0)	0.534
	*n* (%)	*n* (%)	*n* (%)	X^2^; *p*
Nintedanib (*n*/%)	20 (27.7)	21 (39.6)	12 (22.6)	5.204; 0.074
Pirfenidone (*n*/%)	16 (32.7)	12 (24.5)	21 (42.9)	

Data are expressed as mean ± standard deviation, median (interquartile range], or *n* (%). There is a significant difference between a, b, c groups. Abbreviations: ALI: advanced lung cancer inflammation index; GAP: gender, age, and two lung physiology variable index; FVC: forced vital capacity; DLCO: diffusion capacity for carbon monoxide; 6MWT: six-minute walk test; BMI: body mass index; NLR: neutrophil/lymphocyte ratio.

**Table 3:** Relationships between simple blood cell count indexes and clinical parameters.

		Gap Stage	FVC	DLCO	6MWT
ALI	r	−0.815	0.498	0.637	0.445
	*p*	0.000	0.000	0.000	0.000
BMI	r	−0.634	0.406	0.493	0.499
	*p*	0.000	0.000	0.000	0.000
NLR	r	0.638	−0.348	−0.525	−0.257
	*p*	0.000	0.000	0.000	0.009
Albumin	r	−0.636	0.410	0.431	0.412
	*p*	0.000	0.000	0.000	0.000

Abbreviations: BMI: body mass index; GAP stage: gender, age, and two lung physiology variable index; FVC: forced vital capacity; DLCO: diffusion capacity for carbon monoxide; NLR: neutrophil/lymphocyte ratio; ALI: advanced lung cancer inflammation index; 6MWT: six-minute walk test.

**Table 4:** ROC curves for albumin, BMI, ALI, and NLR in predicting mortality in IPF patients.

	AUC (%95)	Cut Off	*p*	Sensitivity (%)	Specificity (%)
Albumin	0.952 (0.904–1.000)	2.45	0.000	63.6	97.8
BMI	0.885 (0.811–0.959)	21.84	0.000	54.5	94.5
ALI	0.945 (0.892–0.998)	11.20	0.000	63.6	98.9
NLR	0.768 (0.600–0.936)	5.25	0.000	36.4	98.9

Abbreviations: BMI: body mass index; NLR: neutrophil/lymphocyte ratio; ALI: advanced lung cancer inflammation index; ROC: receiver operating characteristic; AUC: area under the curve; IPF: idiopathic pulmonary fibrosis.

## Data Availability

The datasets used and/or analyzed during this study are available from the corresponding author upon reasonable request.
